# The Engineering Side of Hospital Work

**Published:** 1905-02-11

**Authors:** 


### THE ENGINEERING SIDE OF HOSPITAL WORK.

*Continued from page* 340.

### III.—COLD STORAGE AND REFRIGERATION.

### THE MECHANICAL PRODUCTION OF LOW TEMPERATURES.

As mentioned in the last article, the mechanical production of low temperature is accomplished by making use of the facility with which ammonia ($NH_3$), carbonic acid ($CO_2$), and sulphurous acid ($SO_2$), can be transformed from the gaseous to the liquid condition, and *vice versa*. All mechanical refrigeration apparatus contain a condenser, in which the gas is converted to the liquid form; a compressor, in which the molecules of the gas are brought closer together before passing to the condenser, and an expansion apparatus, in which the liquefied gas is allowed to return to the gaseous form. The expansion apparatus, usually called the expansion coil, is placed in the neighbourhood of the substance from which it is desired to extract heat; the liquefied gas in expanding absorbing from its immediate neighbourhood the heat to enable it to assume the gaseous condition. In some cases, therefore, the expansion coils are placed in the tank in which ice is to be made, or in the chamber it is required to keep at a certain temperature. More frequently, however, the expansion coils are enclosed in a tank containing brine, made by the solution of calcium chloride in water. The heat required for enabling the liquefied gas to assume the gaseous condition is abstracted from the brine surrounding the coils, and the brine is thereby cooled, and is pumped to the chamber to be cooled, or the tank in which ice is to be made, returning to the expansion tank after it has performed its work in cooling the air of the chamber, or the water from which ice is to be made, hotter than when it left the tank. It is cooled by again assisting some portion of liquefied gas to assume the gaseous condition, is again pumped to the chamber, or the ice tank, and so on.

### THE REFRIGERATING CIRCUIT.

The refrigerating circuit is as follows. The gas, in a liquid condition, is allowed to pass into the expansion coils, where it assumes the gaseous condition. Having done its work in the expansion coils it is sucked back to the compressor, where its volume is reduced, and it then passes to the condenser, where it assumes the liquid form, thence passing to the expansion coils, and so on.

### THE COMPRESSER.

Compression is accomplished in two distinctly different ways, which are termed respectively the compression and the absorption systems. Both are really compression systems in the sense that both compress the hot gas, but in the compression system there is a cylinder with a piston working in it, into which the gas is sucked from the expansion coils, and where the piston, on its return stroke, causes the molecules of the gas to close up together, while in the absorption system advantage is taken of the well-known property of water for absorbing gases, and of the fact that the ability to absorb gas lessens as the temperature of the solution increases, to compress the gas, much as the pressure of steam is raised in a steam boiler. In the absorption system the returning gas from the expansion coils passes into a vessel containing a weak solution of the gas in water. It should be mentioned that the absorption system has, so far, only been applied to ammonia ($NH_3$). The liquid which has absorbed the gas, when it attains a certain saturation, is pumped into a vessel called the generator, which is heated by steam passing through the vessel in pipes. The temperature of the solution being raised, its capacity for holding the gas in solution is reduced, and the gas comes away, very much as steam does in a steam boiler. The pressure of the continually generated gas causes some of the gas to be continually passing to the condenser, just as with the cylinder compressor and piston. In both cases there is a continuous passage of gas from the expansion coils to the compressor apparatus, and a continual passage of compressed gas from the compressor to the condenser. With the absorption process the operation of compression is accomplished in two sections, while in the cylinder compressor it is accomplished in one. There are other apparatus in connection with the absorption system arranged to ensure increased economy that will be described later. Meanwhile we pass to

### THE CONDENSER.

There are two forms of condenser, but both are on the same principle. The office of the condenser is to deprive the gas of the heat which enables it to exist as heat, and of the heat which it has acquired in the operation of compression, and the process in both forms is, the heat is transferred to water provided for the purpose. The two forms are the submerged condenser and the atmospheric condenser. In both the hot gas passes through the apparatus in lengths of pipe, over which water is continually passing, the heat from the gas passing to the wall of the pipe, and thence to the water. In the submerged condenser the pipes in which the gas is passing are enclosed in a tank, through which a stream of water is kept in continual circulation. In the atmospheric condenser the pipes are exposed in the open air, and the water trickles down over the surface of the pipes. The valuable well-known property of extraction of heat by the evaporation of water is made use of in this apparatus. A portion of the water which trickles down over the pipes evaporates, and in doing so demands heat to enable it to assume the vaporous condition, the heat required being abstracted from the cooling water itself, the pipe and the gases passing in them. It will be seen that the heat taken from the cold chamber is passed to the gas, and thence to the cooling water, so that the problem involved is the transportation of the heat to the cooling water, and thence to the drain or the atmosphere.

### COOLING THE COOLING WATER.

Where water is scarce or dear it is often advisable to use the cooling water of the condenser over and over again as far as this can be done, and this is accomplished by different methods, all of them modifications of the principle of the atmospheric condenser. The warm water which has done its work in the condenser is taken to the top of a tower and allowed to trickle down, meeting a stream of air as it descends. A small portion of the water is evaporated, the heat necessary being abstracted from the water itself to a large extent, and the water itself cooled. One important feature of this is the warmer the air that meets the water to be cooled, the greater is the cooling effect. At the Savoy Hotel the warm air from the kitchens, boiler-house, etc., is used to cool the condensing water with complete success.

### COMPRESSOR REFRIGERATION PLANT.

In compression plant the different parts of the apparatus are combined together as far as possible. In small plants it is usual to fix the condenser and the expansion tank in one. A cylindrical or rectangular tank is supported on a bed-plate in the usual way, and the outer surface of the tank forms the bed-plate for the compression cylinder, for the bearings of the crank shaft, the pump, and other accessories. The tank itself is divided into two portions by a double partition, insulating material being placed between the two partitions so that the two divisions of the tank are thermally insulated from each other. In one division the condenser pipes are fixed, with a continuous stream of water passing through the division and around the pipes. In the other division the expansion pipes are fixed, with a stream of brine constantly passing through the division and around the pipes. The brine, as already explained, is pumped to the brine-grids in the cold chamber or in the ice-tank, returning to the expansion-tank after it has done its work, to be recooled and so on. The compressor may be driven by a small steam cylinder, which in that case is carried also on one face of the combined tank or by an external source of power, as an electric motor, and in that case a crank shaft is provided, running in bearings fixed on the face of the tank, and having a pulley for taking the power from the motor. A gas or oil engine, or a belt from a running shaft used for other purposes may also be employed. The pumps for the circulating water and the brine are also carried on the face of the tank. In some cases, even with small plants, the expansion tank is kept separate from the condenser, and it is always so in large plants. The condenser is usually arranged, however, to form part of the complete apparatus so as to economise space. A frequent arrangement is to fix the condenser in the under part of the casting supporting the driving engine and compressor, where these are horizontal.

Auxiliary Apparatus, With the Absorption System.

In the absorption system, as explained, the gas is alternately absorbed by the water and expelled from it, but the working of the arrangement is not quite so simple as this. In the process of absorption of the gas heat is liberated, the gas being reduced to the liquid form in the process of solution and giving up its latent heat to the solvent. This heat has to be removed as it tends to decrease the capacity of the solvent for absorbing the gas. Further, when the gas is driven off by the heat delivered to the solution by the steam, small quantities of watery vapour come away with the gas, just as it does in a steam boiler, and this has to be got rid of, or the efficiency of the apparatus will be lowered and the working will be interfered with. Again, as the solution in the absorber becomes more and more saturated and that in the generator less so, the strong and weak liquors separate owing to their different specific gravity, and this is taken advantage of to keep the generator, where the gas is being disengaged, continually supplied with strong liquor. There is a continual passage of strong liquor from the absorber to the generator and of weak liquor, liquor that has been deprived of its gas, from the generator to the absorber. But these liquors are in the wrong condition thermally. To absorb the gas the liquor must be at as low a temperature as possible, the lower the temperature the more it will absorb, while on the other hand the generator requires to be at the highest possible temperature, the higher the temperature the better will the gas come away. Hence an apparatus called an exchanger has been added. The exchanger is a cylindrical vessel containing pipes, and arranged so that while the liquor from the absorber passes in the pipes, the liquor from the generator passes around them, the hot liquor from the generator giving up some of its heat to the liquor from the absorber, the latter passing on to the generator at a higher temperature and the former passing on to the absorber at a lower temperature. The liquor on its way to the absorber is also cooled by a stream of water which has already done duty in the condenser and another apparatus. There are yet two other apparatus, the analyser and the rectifier, both of which are engaged in separating the watery vapour from the gas. The analyser usually forms part of the generator and consists of baffle plates, which arrest some of the watery vapour as it ascends from the surface of the hot solution and causes it to condense, the condensed vapour falling back into the solution. The rectifier is an apparatus somewhat similar to the generator. It consists of a cylindrical vessel, through which the gas and vapour pass. A stream of cooling water passes through pipes fixed in the cylinder, the gas and vapour passing round them. The mixture of gas and water vapour being cooled, the water condenses and is carried back to the generator, the gas passing on to the condenser. The complete apparatus, as made by Messrs. Ransome and Rapier, who are almost the only makers of absorption machines in this country, consists of several iron cylinders, two of which are fixed side by side on the floor and the rest vertically one above the other, the generator being at the bottom and condenser at the top. A stream of water passes through the condenser, the rectifier, and the exchanger, cooling each on its way, a small pump fixed on the top of one of the cylinders keeping the water in circulation. Steam circulates in pipes in the generator furnishing the heat required. The process is quite continuous. The expansion coils are usually away from the apparatus, in a brine tank or wherever cooling is wanted, a separate pump keeping the brine in circulation. The gas is maintained in circulation, as already explained, by its creation in the generator, and the process is rendered continuous by the exchanging and other apparatus described. In the absorption apparatus the generator starts the action with a charge of ammonia gas in solution. The heat furnished by the steam drives some of the gas over to the rectifier and thence to the condenser, whence it passes to the expansion coils, thence back to the absorber, and thence in solution back to the generator again, and so on. The solution in the generator is gradually weakened by the delivery of the gas, that in the absorber gradually gets stronger, and the exchange mentioned keeps the balance working.

